# Macrophage Identification In Situ

**DOI:** 10.3390/biomedicines9101393

**Published:** 2021-10-04

**Authors:** Krisztina Nikovics, Anne-Laure Favier

**Affiliations:** Imagery Unit, Department of Platforms and Technology Research, French Armed Forces Biomedical Research Institute, 91223 Brétigny-sur-Orge, France; anne-laure.favier@intradef.gouv.fr

**Keywords:** macrophages, phenotype, in situ hybridization, cytokines, immunolabeling

## Abstract

Understanding the processes of inflammation and tissue regeneration after injury is of great importance. For a long time, macrophages have been known to play a central role during different stages of inflammation and tissue regeneration. However, the molecular and cellular mechanisms by which they exert their effects are as yet mostly unknown. While in vitro macrophages have been characterized, recent progress in macrophage biology studies revealed that macrophages in vivo exhibited distinctive features. Actually, the precise characterization of the macrophages in vivo is essential to develop new healing treatments and can be approached via in situ analyses. Nowadays, the characterization of macrophages in situ has improved significantly using antigen surface markers and cytokine secretion identification resulting in specific patterns. This review aims for a comprehensive overview of different tools used for in situ macrophage identification, reporter genes, immunolabeling and in situ hybridization, discussing their advantages and limitations.

## 1. Introduction

**Figure 1:**
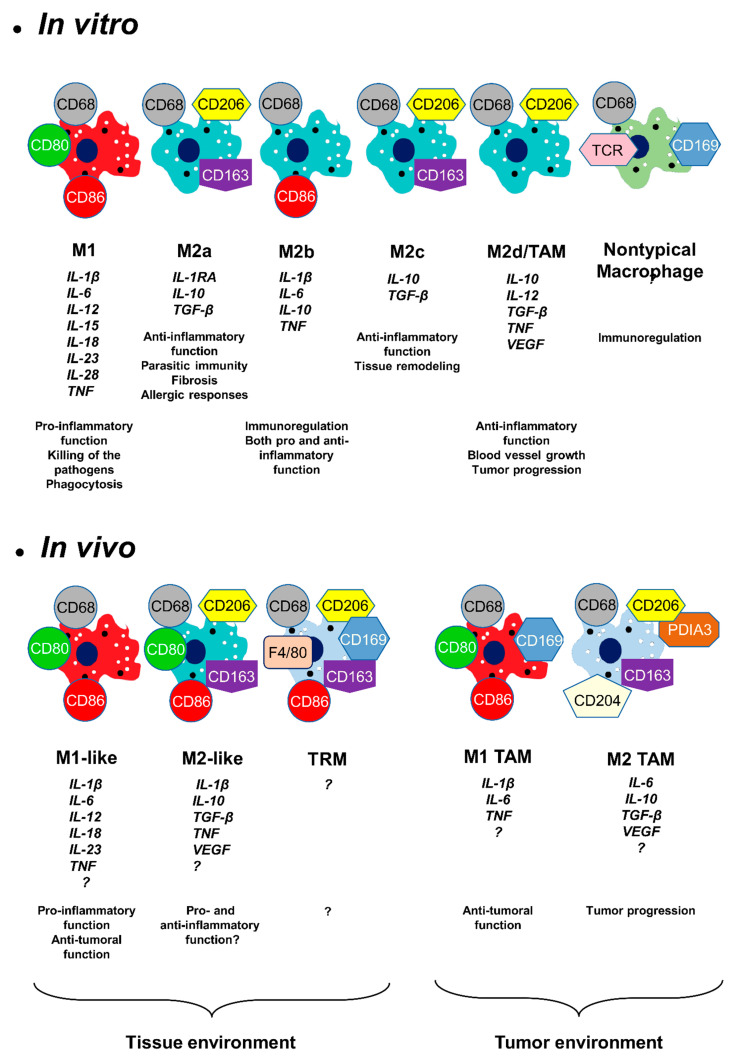
Macrophage polarization sub-types. Different cytokine expressions and functions of the macrophage populations in vitro and in vivo. Tissue Resident Macrophage (TRM), Tumor Associated Macrophage (TAM).

In the late of 19th century, Elie Metcnikoff described macrophages for the first time and hypothesized that these mononucleated phagocytic cells might play an important role in the immune response [1]. Later, it was discovered that macrophages have indispensable function during innate and adaptive immunity. Tissue injury promotes a series of well-coordinated events, which contribute to the efficient healing of damaged tissue. Reduction in inflammation and improvement in tissue regeneration is very important. Macrophages perform an essential function in both processes [2]. In the absence of macrophages, poor tissue healing, imperfect angiogenesis and fibrosis appears [3], resulting in an inhomogeneous population of cells detectable in every tissue of the body depending on the stimulus and the environment in which they are located [4,5,6,7,8,9]. The polarization of macrophages is determined by many biological signals (growth factors, fatty acids, prostaglandins, and pathogen-derived molecules) in addition to cytokines located in the micro-environment. Macrophages have critical biological roles from inflammation through to resolution and repair. They express and produce various molecules [10] which have different roles during different processes. The view on the characterization of macrophage polarization is rapidly changing [2,11,12]. In the beginning, in vitro culture was used for macrophage phenotype characterizations because, in this situation, the extracellular environment can be easily controlled. In the 1990s, it was discovered that lipopolysaccharide (LPS), interferon (IFN)-gamma and cytokine IL-4 can induce the expression of different genes in macrophages. Two major groups were distinguished during polarization. Macrophages activated by LPS were named as “classically activated macrophages” and the IL-4 stimulated macrophages were named as “alternatively activated macrophages” [11,13,14]. One hundred years ago, Otto F. Warburg discovered that activation of macrophages modified their glucose metabolism and increased lactate production [15,16]. Aside from glucose metabolism, amino acid metabolism can also be altered in the classically activated macrophages [17]. LPS/IFN–gamma stimulation induces arginine conversion to nitric oxide (NO). This molecule has a cytotoxic and bacteriostatic effect [18]. However, the IL-4 inhibits NO production in the alternatively activated macrophages [19]. In addition to these metabolic pathways, alteration of the tricarboxylic acid cycle was also observed during alternative macrophage activation. High levels of succinate triggers intense inflammation in the classically activated macrophages [17]. Mills and colleagues (2000) proposed a new classification of the macrophages (M1 and M2), and the scientific community decided to name the classically activated (pro-inflammatory) macrophages M1 and the alternatively activated (anti-inflammatory) macrophages M2 [20]. In vitro results showed evidence of new M2 macrophage sub-types (M2a, M2b, M2c with different functions and expressing different molecules (Figure 1)) [21,22].

The M1/M2 classification is not perfect because particular macrophages do not fit into any of the sub-types. These are the nontypical macrophages. Some produce T cell receptor (TCR) or CD169 protein and they do not promote phagocytosis but are involved in immune regulations [23] (Figure 1).

In 1988, the growth arrest-specific gene 6 was discovered [24]. This protein binds to the TAM (Tyro3, Axl and Mer) receptor family and plays an important role in the development of different cancers [25]. Qian and Pollard (2010) have suggested that the distribution of M1 and M2 macrophages is more complex, as tumor-associated macrophages (TAM macrophages) showed a different pattern compared to M1/M2 criteria [26]. Finally, TAM macrophages were classified as a new sub-type, the M2d macrophages [27]. Later it turned out that the TAM macrophage phenotype is even more complex, finally splitting into M1 and M2 TAM macrophages. M1 TAM macrophages are involved in anti-tumorous activity while the M2 TAM macrophages induce the tumor progression and metastasis formation [28,29,30,31] (Figure 1).

In that way, the in vivo landscape is more intricate compared to the in vitro situation [32,33,34,35,36,37]. The in vivo macrophages are generally called M1-like and M2-like or resolving macrophages [3]. Due to purification cell process, various immune cells are absent from in vitro environment studies, resulting in a lack of cytokine expression which is usually involved in macrophage polarization in vivo. In this context, the characterization of in vivo macrophages is still incomplete. Thus, many laboratories are interested in the identification and characterization of M1-like, M2-like and tissue-resident macrophages. An increasing number of articles have pointed out that contrary to the original theory, in vivo macrophage phenotypes offered a more complex pattern of cellular markers and cytokine expression panel [3,38] (Figure 1). Various publications suggested that in vivo M2-like macrophages exhibited mixed pro- and anti-inflammatory functions [9,21,35,39,40,41,42].

Similarly, the precise function by which tissue-resident macrophages contribute to tissue regeneration is currently not fully understood, but they also play an important role in the process of wound healing (Figure 1) [3,43,44,45]. They contribute to maintaining homeostasis by constantly monitoring the internal and external signals within the body. After injury, the distinct secreted signals help to restore homeostasis. In the adult mouse, the tissue-resident macrophage populations originate equally from embryonic precursors and from bone marrow monocytes [46]. Orecchioni and colleagues (2019) have suggested, however, that in mice a majority of tissue macrophages are not monocyte-derived and mature tissue macrophages derived from embryonic precursors. This point explains why certain marker proteins used to characterize macrophages in rodent cells are not appropriate for human macrophage identification and vice versa [34].

The in vitro and in vivo macrophages produce a wide range of secretory molecules (proteinases, chemokines, pro- and anti-inflammatory cytokines, growth factors and metabolites derived from oxygen, nitrogen, arachidonates) [11]. Specific macrophage phenotypes express different cytokines (Figure 1). In vitro M1 macrophages express pro-inflammatory cytokines, such as tumor necrosis factor (TNF), interleukin-1 beta (IL-1β), interleukin-6 (IL-6), interleukin-12 (IL-12), interleukin-15 (IL-15), interleukin-18 (IL-18), interleukin 23 (IL-23) and interleukin 28 (IL-28). The M2a phenotypes produce anti-inflammatory cytokines, such as interleukin-10 (IL-10), interleukin-1 beta-receptor antagonist (IL-1RA) and transforming growth factor beta (TGF-β). Surprisingly, the M2b phenotypes express pro-inflammatory cytokines (TNF, IL-1β and IL-6) and anti-inflammatory cytokines (IL-10). The M2c phenotypes produce exclusively anti-inflammatory cytokines IL-10 and TGF-β. Finally, the M2d subtype expresses also both pro-inflammatory (TNF and low level of IL-12), anti-inflammatory (IL-10 and TGF-β) and proangiogenic cytokines (vascular endothelial growth factor (VEGF)). Cytokine expression pattern of the in vivo macrophages have not yet been fully discovered (Figure 1) [3,4,5,6,8,9,33,42,47].

**Figure 2:**
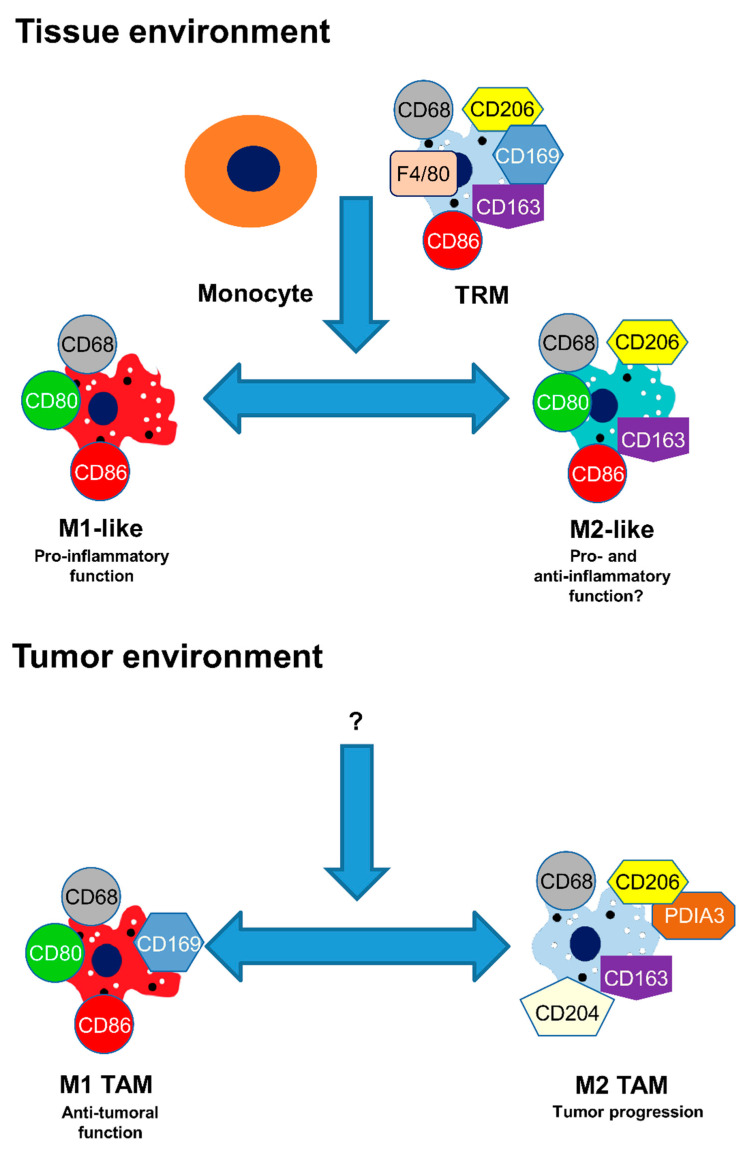
In vivo macrophage plasticity. Tissue Resident Macrophage (TRM), Tumor Associated Macrophage (TAM).

Polarization is a complex process. When un-activated macrophages (M0) in vitro are stimulated by LPS, they can undergo phenotypical and functional changes and transform into M1 macrophages [5,12,21,36]. Analysis of human macrophages showed that activation by IL-4/IL-13 induce the switch to the M2 phenotype of the macrophages. However, some other processes may be involved because mouse wounds do not contain these cytokines [48]. Moreover, under an environmental change, M2 macrophages may reversibly revert to the original M1 polarization [49,50]. Plasticity is an essential function of macrophages and plays an important role in the development of various diseases and cancer [7,33,51]. In vivo, monocyte and tissue-resident macrophages are also capable of polarizing to the M1-like macrophage phenotype and, after different environmental conditions, to the M2-like macrophage. In addition, stimulation of human M1-like macrophages with an IL-13 cytokine results in the transformation of such M2-like macrophages that gained phagocytic activity [7,52,53]. M2-like macrophages can easily revert to M1-like macrophages. These cells lose their endocytic but not their phagocytic activity [54]. In the tumor environment, large amounts of leucocytes are obtainable, most of which are TAM macrophages [36]. These cells play an essential role in the relationship between inflammation and cancer. In the initiation stage of the cancer, M1 TAM are located in the tumor environment and during tumor progression, they switch into M2 TAM macrophages. Anti-tumor molecules can cause M2 TAM to revert to M1 TAM macrophages (Figure 2) [26,55,56,57].

In order to characterize macrophages, investigating the pattern of cytokine expression together with cell surface markers is essential. Diverse techniques are available, some of which focus on gene expression analysis, such as Northern blot, qPCR, microarray, flow cytometry and next-generation deep sequencing methods [58]. The limitation of these techniques is that a signal results from a mixture of diverse cells [59].

To determine the in vivo macrophage phenotypes in situ, it is crucial to identify the spatial resolution of the cytokine and cell surface markers expressing cells in the morphologically well-conserved tissue. To carry out this analysis, three methods are available: (i) expression of a reporter gene; (ii) classical immunolabeling techniques using antibodies against specific surface protein markers and cytokines; (iii) in situ hybridization (ISH) methods.

Macrophages can play both protective and pathogenic roles in different diseases. The polarization of these cells is not fixed and the outcome depends on the signals they receive in a given place and time. Therefore, investigation of the in vivo macrophage phenotypes in situ is fundamental to improve our understanding of tissue regeneration. This review provides an overview of in situ tools used for macrophage characterization.

## 2. Tools for Labeling Macrophages In Situ

During tissue regeneration, distribution of the M1-like and M2-like macrophages is important [60,61] to better understand the involvement of macrophages in the biological process. We then focused on tools for labeling macrophages, and several in situ approaches in macrophage biology will also be discussed (Table 1).

### 2.1. Reporter Genes

Fluorescence molecules are genetically encoded proteins. For in vivo or in vitro expression analysis, a fusion between a target protein and a fluorophore was used [62]. In 1962, the wild-type green fluorescent protein (GFP) has generated as a marker for gene expression and localization from the jellyfish *Aequorea victoria* [63]; soon after, two GFP mutants with brighter fluorescence, S65T and EGFP (F64L/S65T) were discovered. EGFP became one of the most widely used reporter molecules [64]. The huge advantage of EGFP compared to other reporter molecules was that this protein could be detected directly in living cells, and no specific treatment was needed for protein analysis [65]. The original GFP emitted green light. Subsequently, colorful variants of the GFP also appeared (BFP (blue); CFP (cyan); YFP (yellow)), producing fluorescence in different colors [66,67]. The next types were the red fluorescent proteins, such as DsRed from *Discosoma* and HcRed from *Heteractis crispa* [68,69], but they suffered several problems because of their toxicity and they tend to form tetramers. After unceasing progression, other orange and red fluorescent proteins were developed with better spectral properties, such as tdTomato and mCherry, respectively [64,70]. In the early 2000s, other fluorescent reporter molecules became available and eventually the fluorescence emission profile covered the entire visible light spectrum [64,71,72]. In living cells, fluorescent proteins are the most common fluorescent molecules. However, their big size is a disadvantage that can affect the properties of the target protein [62].

Determining localization and topology of the macrophage markers within the cell is essential to characterize macrophages. In order to answer this question, various reporter gene fusions with specific macrophage marker promoters or macrophage marker proteins were engineered. One approach to studying macrophage differentiation in vivo was the use of transgenic mice expressing GFP under the control of the human Cluster of Differentiation (CD) *CD68* promoter intron1 [73]. This method is extremely useful for directly monitoring the migration of monocytes from the bone marrow to the tissue and for visualizing their transformation into macrophages and differentiation during inflammation/regeneration. This tool could be a powerful resource in macrophage biology to study the process of inflammation and healing. However, it requires animal facilities and agreement to work with transgenic animals for the analysis and expression of the *CD68* promoter-intron1/GFP transgene, and it showed some differences to the endogen macrophage marker [73,74].

Another helpful approach for in situ analysis of macrophages may be the use of bacteria containing the fluorescence reporter gene [75]. The use of such bacteria can provide important information on the details of the bacteria–macrophage interactions [6]. Fluorescence markers containing bacteria can contain promoter-reporter genes or bacterial gene-reporter gene fusion constructs. The second type of bacteria can provide not only information on the protein localization but also about the bacterial replication status in situ [76].

Tissue-specific macrophages form a heterogeneous population. De Schepper and colleagues (2018) have demonstrated the difference between self-maintaining and monocyte-originated macrophages [77]. A special group of tissue macrophages is microglial cells of the central nervous system (CNS) [78]. These cells have recently become a focus of interest, as they have an important function during CNS neuroinflammation and disease [79]. Different mouse reporter lines were used, and most of them bear the fractalkine receptor gene *Cx3cr1* [80]. However, this gene is expressed not only in microglia but also in other immune cells [79]. Nowadays, a new generation of microglial mouse reporter lines has been generated in which the reporter protein is mostly restricted to microglial cells [81,82,83]. These transgenic animals provide essential information about neuroinflammation.

Tumor microenvironment demonstrated immune cell penetrations. TAM macrophage played an important role in all steps of tumor development [84]. TAM macrophages could be promising target cells for cancer therapy. However, more information on these cells, and therefore in situ characterization of TAM macrophages, is fundamental [85]. Several laboratories are using fluorescence proteins in tumor research areas to detect the interaction between TAM macrophages and cancer cells [71,86,87].

### 2.2. Immunolabeling

In situ visualization of macrophages remains one of the most challenging tasks, and immunolabeling is widely used to this effect. One of the most common pan-markers to label monocytes/macrophages is the CD68 protein [45,49]. CD68 is a well-known glycoprotein, intensively expressed by macrophages. However, CD68 can also be detected in other mononuclear phagocyte cells. Moreover, a weak expression can also be seen in other non-hematopoietic cells (mesenchymal stem cells, fibroblast, endothelial and tumor cells) [88].

The mouse is frequently used as an animal model in clinical research, but unfortunately, in many ways, mice differ from humans [78]. Indeed, rodent and human in vitro macrophages can produce different protein over-stimulations, which may potentially indicate diverse functions [11]. The second macrophage pan marker, successfully used in situ in human cells, is the CD11b protein [45].

The polarization of macrophages is a complex process that is highly dependent on the tissue environment [7]. Using this approach, various studies aimed at finding key markers to distinguish between M1-like and M2-like macrophages [7].

It is widely accepted that the phenotypic M1-like macrophage markers are co-stimulatory molecules, such as CD80 and CD86 in mice [49]. Some differences need to be mentioned between human and rodent macrophage marker gene expression. Mouse macrophage produces F4/80 protein (also known as Epidermal Growth Factor-like module-containing mucin-like hormone receptor-like 1) [89]. Baud and colleagues (1995) cloned the human homolog of the *F4/80* gene, the sequence homology between two genes was 68% [90]. Surprisingly, human macrophages did not produce this protein [91]. Other proteins (Ym1, Fizz1 and arginase-1) can also be detected in mouse M2 macrophages but are lacking in human M2 macrophages. [45,92]. Little information is currently available on macrophages in mammals other than rodents and humans. Consequently, it is essential to study another animal model with closer similarity to humans. The progression of diseases in pigs is similar to that in humans at metabolic and infectious levels, which makes mini-pigs an ideal model for macrophage characterization [93,94,95]. In the mini-pig model, under in situ conditions, CD80 protein is not able to accurately distinguish between M1-like and M2-like cells, since M2-like cells also produce CD80 protein [60]. Moreover, intensity of CD80 protein expression in cells depends on both the quality and the quantity of the stimulus [54]. In addition, both CD80 and CD86 expression can also be detected in other cells, such as dendritic cells (DCs), B cells and T cells [96,97] while CD86 can be detected in human M2a-like macrophages [7].

Markers such as CD163 and CD206 generally recognize M2-like macrophages. CD206 protein is a commonly accepted M2-like macrophage marker in rodent and human cells [7]. However, this protein is also expressed at low levels in satellite cells [45]. Another limit is that, depending on the anti-CD206 antibody used, different results can be obtained [98]. The CD163 is considered as an M2-like macrophage marker in human [7] and murine cells [99], but also in the human dendritic cells [100]. In situ detection of human and mouse M2 macrophages is possible by the double staining immunolabeling technique using mannose receptor C type 1 (MRC1/CD206) or CD163 combined with CD68 antibodies together.

Up to now, it is assumed that the M1-like and M2-like macrophages have a monocyte origin [34]. Ginhoux and Guilliams (2016) reported that, in mice, the tissue-resident macrophages are of embryonic origin and persist in the tissue after cell division [101]. The role of tissue-resident macrophages in inflammation and tissue regeneration is not yet fully understood. The only exception is the microglia, which are the specialized tissue-resident macrophages of the central nervous system (CNS). This cell type has recently been intensively studied because of their essential function during inflammation, infection and response to injury [79]. Microglia cells produce proteins that can be detected in the peripheral myeloid cells (CD68, CD45, CD11b, F4/80, Cx3cr1 and CSF1R) [102]. The aim of various studies was to find a specific microglia marker not produced in peripheral myeloid cells. Traditionally, Iba-1 was the microglia pan marker; however, this protein was also expressed in other myeloid cells [103,104]. Later, Bennett and colleagues (2016) showed that both mouse and human microglia cells generate Tmem119 protein for which no expression in peripheral myeloid cells was detected [105]. Yet, another group has shown that the expression of this protein was not restricted to microglia cells [106]. Butovsky et al. (2014) showed that antibodies produced against P2ry12 and FCRLS proteins could make a difference between microglia and infiltrating myeloid cells [107].

In the past, tissue-resident macrophages were identified by morphological analysis or histological staining [108]; the development of various antibodies enabled the detection of macrophages. In mouse animal models, one particularly useful antibody was the anti-F4/80 antibody. F4/80 protein is a cell surface receptor selectively expressed on murine macrophages. The F4/80 protein is a useful positive control in murine macrophage biology because it is a well-resistant protein during the fixation process [46,89]. However, the very heterogeneous expression level of the F4/80 protein in the different tissues could be a disadvantage [109]. In situ conditions make it difficult to distinguish between M2-like and tissue-resident macrophages once they co-exist in a common environment, as there is no good marker gene to make a difference. Gut tissue-resident macrophages produce CD169 proteins [43,77]. However, this protein can also be detected in TAM and in nontypical macrophages [23,110].

TAM macrophages contribute to the initiation and progression of tumor development [27]. It is usually thought that TAM macrophages originate from blood monocytes [111], but in certain brain and lung (murine model animal) cancers, the tumors originated from tissue-resident macrophages [28]. M1-like TAM macrophages express CD68, CD80, CD86 and CD169 [110,112] and the M2-like class of TAM macrophages show strong CD204, CD206 and CD163 expression [112,113,114]. In addition, a new marker for these cells was recently found, the disulfide-isomerase A3 (PDIA3) receptor [115].

Immunolabeling is an excellent method for protein detection in situ. It has to be mentioned that the expression patterns differed depending on the antibody used [98]. Moreover, appropriate protocols need to be developed to obtain a positive signal. Careful signal analysis has to be performed as nonspecific labeling can result from hydrophobic interactions of proteins, ionic and electrostatic interactions, avidin and biotin, Fc receptors or autofluorescence [61,116]. In addition, specificity of the antibody can be limited, as the peptides recognized by antibodies are small (6–10 amino acids) and this antigen can be found in other proteins [117].

### 2.3. In Situ Hybridization

In situ hybridization (ISH) was invented simultaneously by Gall and Pardue (1969) and by John and colleagues in 1969 [118,119]. The name “in situ” is derived from the Latin phrase for “in position”, and hybridization means hybrid formation between different molecules. The various ISH techniques in macrophage biology are summarized in Table 2. The idea of this technique is that RNA and DNA molecules can form a hydrogen bond hybrid based on the complementarity of RNA or DNA sequences. In this approach, differently labeled RNA or DNA probes make a hybrid molecule with the mRNA of interest. ISH allows a very powerful analysis of de novo gene expression because it is highly specific and shows the mRNA localization in the tissue environment. The disadvantages of this technique are the complex probe design and the fastidious steps, which require some optimization for each probe and tissue [120]. An additional challenge is the conservation of the RNA because RNase enzymes are present everywhere,: on glassware, in reagents, on clothes and hands [121]. In the beginning, radioactive probes were used because radioisotope-labeled probes are more sensitive than non-radioactive probes. This hybridization can be detected by autoradiography. After hybridization with the radioactive probe, the sections are coated with an emulsion containing silver ions. The energy liberated from radioactive molecules can transform the silver ions to metallic silver, and this reaction results in image formation [122]. This technique was used to demonstrate that infection induces lysozyme expression in macrophages [123] and that VEGF induces the macrophage recruitment [124]. This method was also utilized for hepatitis A viral RNA detection in macrophages [125]. Unfortunately, a long exposure requirement and harmful effect on health drastically reduced this practice [126].

The next method replacing the radioprobe–ISH method was the Chromogenic In Situ Hybridization (CISH). The digoxigenin-labeled probe is commonly used for CISH in macrophage biology [60,61,98,127,128]. The CISH method exhibits several improvements compared to the radioactive probes, such as better detection, shorter manipulation, good repeatability, easier visualization and being less harmful to health [120]. In the 1980s the Fluorescence In Situ Hybridization (FISH) method appeared [129], which was recently used for microglial signature gene analysis of the mouse brain during development and neuroinflammation [130,131].

Both techniques are based on the same principle, probe hybridization to the mRNA of interest, then detection by an appropriate system [132]. The main differences between the two methods are: (i) in the CISH, the detection is carried out by an enzymatic reaction, the signal intensity does not decrease over time but, unfortunately, only a single expression can be tested in one step. To test two different mRNA productions, substantial time is required, which makes the technique complicated. Enzymatic detection generates a colored precipitate at the site of hybridization. Two enzymes, alkaline phosphatase (AP) and horseradish peroxidase (HRP), are generally used in this method. Although these enzymes can directly be attached to the nucleic acid molecule, the enzyme-coupled probes have very little incorporation property. Therefore, an indirect method is usually used during hybridization, which makes this method lengthy and cumbersome [121]. (ii) In the FISH method, several fluorescent molecules can be used for detection, then multiple mRNA expressions can be analyzed in the same sample, but the signal intensity decreases over time [133] and only highly abundant mRNA can be localized. In this technique, fluorophores can be linked directly to the nucleic acid probe, and after hybridization this fluorescence is directly detectable (the method called “direct labeling”). In a second method (called the “indirect method”), the non-fluorescent molecule is linked to the probe, and this molecule can be visualized by the fluorescence molecule [134].

Characterizing in situ macrophage sub-types is not an easy task because there is no specific marker for one sub-type; therefore, characterization is based on their pattern of both cytokines and cell surface proteins (Figure 1). Two difficulties have to be noted: i, the FISH approach is not a sensitive tool, thus requiring a strong enough cytokine expression level to be detected. ii, cytokines are secreted proteins, making it complicated to link the signal with the macrophage source of expression by immunolabeling [60]. Recently, a new revolutionary in situ hybridization, the Hybridization Chain Reaction (HCR), has emerged [135]. HCR is an isothermal enzyme-independent nucleotide polymerization method. The idea of this method results in the use of two hairpin oligonucleotides linked with fluorophore. When the complementary initiator nucleotide hybridizes with mRNA, the initiator activates the hairpin molecules, and they assemble into a well-defined structure providing a source of fluorescence. The main advantage of this method is that it is very sensitive and therefore suitable for the detection of the low abundant mRNAs [136,137,138,139,140]. This new approach was used for identification of macrophage sub-types by analysis of cytokine expression. This technique allowed the in situ visualization of M2d-like macrophages in the mini-pig model [60].

Another powerful technique that allows the localization of RNA to specific cells is the RNAscope^®^Multiplex Fluorescent Assay V2 (Advanced Cell Diagnostics, Newark, CA, USA). This technique uses paired double-Z oligonucleotide RNA probes for hybridization. These oligonucleotide probes contain several linker sequences. The amplifiers and the color label probes are sequentially added to the linker sequence. It is highly sensitive, and multiple labeling can be performed on the same tissue section. This technique was used as a prognostic indicator in human kidney cancer. In certain cancers, the von Hippel Lindau tumor suppressors are inactivated. Therefore, the HIF-1α produced by M2 TAM macrophages triggers the production of proteins that are required for tumor progression. Detection of this protein by in situ hybridization can then provide important information about tumor development [141]. This method is, however, very costly, and therefore less commonly used than other ISH techniques.

### 2.4. Imaging Mass Spectrometry

In recent decades, a great new technique has emerged for in situ imaging, the Imaging Mass Spectrometry [142,143]. With this technique, it is possible to detect hundreds of proteins, peptides or lipids simultaneously in different tissue sections. At the moment, this approach is still less widely used for macrophage characterization [144,145], but it is undoubtedly becoming routine and an essential tool in the coming years.

## 3. Conclusions

During wound healing, macrophages have an essential role. Inflammation and tissue regeneration is complex and can induce well-coordinated gene expression changes in macrophages. For proper tissue healing, the constant interplay between external and internal signals is essential, including cytokine regulation, selective adhesion or import and export of transcription factors to the chromatin. Macrophages localized in the injured area have distinct phenotypes with different functions and they act at diverse times during the reparation. There are several questions that remain to be answered in order to comprehend the repair process, such as what are the precise phenotypes of the in vivo macrophages? To achieve breakthroughs in in vivo macrophage characterization, the development of new tools is essential, as in situ characterization of the macrophages is indispensable to uncover the spatial actors involved in inflammation and tissue regeneration mechanisms. These tools will also provide opportunities to explore macrophage phenotypes in vivo. Indeed, the application of distinct ISH approaches can bring new insights into the polarization of macrophages in situ to investigate tissue inflammation and regeneration.

## Figures and Tables

**Table 1:** Advantages and disadvantages of the different in situ tools.

Tools	Advantages	Disadvantages
Reporter gene	-Protein expression changes can be monitored during development, inflammation or tissue regeneration	-Animal facilities and transgenic animals required -Reporter protein may alter the endogen protein localization -Expression of the recombinant protein can differ from the endogenous protein (level, localization…)
Immunolabeling	-Easy to use -Long signal stability	-Antibody dependent (specificity, sensitivity, animal model…)
In situ hybridization	-High specificity -Detection of various genes sub-type -Viral genome can be detected -miRNA can be detected	-Fastidious -Complex (probe design) -Rnase free equipment needed -Molecular biology expertise required

**Table 2:** Sensitivity, advantages and disadvantages of the different in situ hybridization approaches. ((+) low, (++) medium, (+++) strong sensitivity).

Tools	Sensitivity	Advantages	Disadvantages
Radioactive probe	+++	-Best sensitivity	-Long exposure required -Harmful
CISH	++	-Stable signal	-Cumbersome double labeling
FISH	+	-Multiple labels	-Not stable
HCR	++	-Multiple labels -Increased sensitivity compared to FISH	-Not stable -Probe design strategy
RNAscope^®^ Assay	++	-Multiple labels -Rapid method	-Not stable -High financial cost
